# Revoking a Diploma

**Published:** 1860-10

**Authors:** 


*Revoking a Diploma.*—The N. Y. Medical College has revoked the diploma given by them to a John F. Dunker, of Newark, assigning as a reason that he obtained it under gross deceptions, and that he was a notorious quack. B.

